# Static Characterization of the Driving, Normal and Stall Forces of a Double-Sided Moving-Permanent Magnet-Type Planar Actuator Based on Orthogonal Planar Windings

**DOI:** 10.3390/s18103526

**Published:** 2018-10-18

**Authors:** Marilia A. da Silveira, Marcos J. Susin, Aly F. Flores Filho, David G. Dorrell

**Affiliations:** 1 Post-Graduate Programme in Materials Engineering and Sustainable Processes, Lutheran University of Brazil, Canoas 92425-900, Brazil; marilia.amaralsilveira@gmail.com; 2 Post-Graduate Programme in Electrical Engineering, Federal University of Rio Grande do Sul, Porto Alegre 90690-410, Brazil; marcos.susin@gmail.com; 3 Discipline of Electrical, Electronic and Computer Engineering, University of KwaZulu-Nata, Durbam 4041, South Africa; dorrelld@ukzn.ac.za

**Keywords:** stall force, planar actuator, ironless armature, orthogonal windings, analytical model

## Abstract

This work presents a study of the traction, normal and stall forces in a two-sided planar actuator with orthogonal planar windings and a mover that comprises two cars magnetically coupled to each other through two pairs of permanent magnets (PMs). There is no ferromagnetic armature core because of the permanent magnets array in the mover and orthogonal traction forces can be generated in order to move both cars jointly in any direction on a plane. The stall force is the minimal force necessary to break up the magnetic coupling between the two cars. When one of the cars is subjected to an external force through the *x*- or *y*-axis, the cars can become out of alignment with respect to each other and the planar actuator cannot work properly. The behavior of the forces was modelled by numerical and analytical methods and experimental results were obtained from tests carried out on a prototype. The average sensitivity of the measured static propulsion planar force along either axis is 4.48 N/A. With a 20-mm displacement between the cars along the direction of the *x*-axis and no armature current, a magnetic stall force of 17.26 N is produced through the same axis in order to restore the alignment of the two cars.

## 1. Introduction

A planar actuator can be described as a device that produces movement on a plane by provides movement with a minimum of two degrees of freedom within an area of movement over that plane [1]. Many electromagnetic linear and planar actuator topologies have been studied for different applications. They can be formed by a stationary armature and movable permanent magnets or vice versa. Different kinds of electromagnetic planar actuators have been designed and analyzed in recent studies. The concept of a stationary two-layer coil electromagnetic actuator for planar motion was presented in Reference [1]. An ironless synchronous permanent-magnet planar actuator was discussed in Reference [2]. The study and characterization of the electromagnetic forces produced by linear and planar electromagnetic actuators are based on analytical models generally. In Reference [3], the authors present an overview of analytical models for the design of linear and planar motors. Analytical models for computing the magnetic flux density and electromagnetic forces in linear motors were presented in References [4,5]. In Reference [6], the authors proposed a design methodology for linear actuators; this study considered thermal and electromagnetic coupling with geometrical and temperature constraints. A study of an induction planar actuator concept was discussed and analyzed in Reference [7].

The purpose of this paper is to present the studies on a double-sided moving-PM-type planar actuator based on orthogonal planar windings that form an ironless armature. Its constructive characteristics and principle of operation are presented. An analytical model is proposed in order to analyze the behavior of the involved magnetic forces and of the magnetic flux density vector distribution produced by the permanent magnets and by the current in the windings throughout the actuator. A numerical model of the actuator based on 3D-finite elements was employed as one way to validate the analytical model. Results of measurements are also presented, discussed and compared to the theoretical ones. They provide information about de static performance of the device presented in this paper. Applications such as printed circuit movers and NC systems present potential use for that actuator.

**Figure 1:**
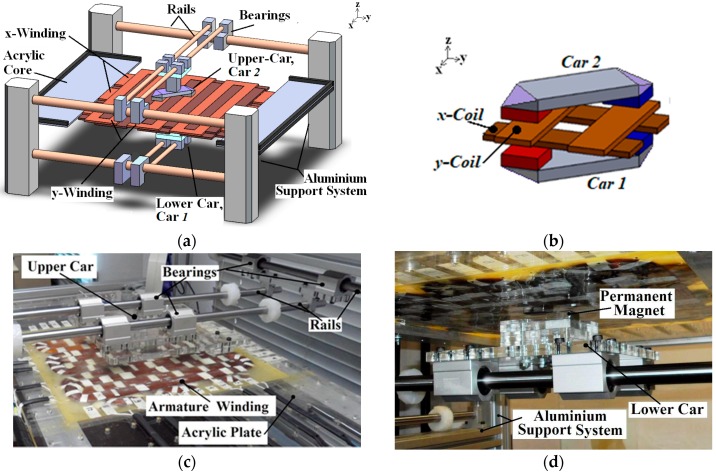
(**a**) Picture of the planar actuator with ironless armature and a double-sided mover [8]; (**b**) details of Cars *1* and *2*; (**c**) the prototype of the device where the upper car (Car *2*) is highlighted; (**d**) the lower car (Car *1*).

The concept of the planar actuator analyzed in this paper was firstly presented in Reference [8]. Figure 1a illustrates its topology. Figure 1b shows details of Cars *1* and *2* where only the coils located in between the cars are presented. Figure 1c,d present a view of prototype of the actuator and its lower car, respectively. The device is based on a double-sided mover that uses two cars magnetically coupled to each other through two pairs of permanent magnets (PMs). An ironless armature with two orthogonal planar windings is placed between the two cars.

The electromagnetic planar actuator has a mover mounted on a mechanical supporting system. The structure employs a set of linear guides and bearings that provides mechanical support for the cars in order to allow bi-directional movement along the *x* and *y* axes. The two cars are magnetically coupled to each other in a symmetrical form that holds them together. The lower car is the magnetic and mechanical mirror of the upper car. Each car has two permanent magnets with opposite magnetization and linked by a steel back iron, therefore forming a complete mover [8].

The armature consists of multiphase windings that are arranged to form two sets of orthogonal windings, that is, a group of six coils or phases assembled along the *x*-axis forming the *x*-axis multiphase winding and a group of six coils or phases assembled on the *y*-axis forming the *y*-axis multiphase winding. This configuration uses an ironless armature since the magnetic flux path crosses the air-gap and the windings in between the two cars to form a closed magnetic circuit with the permanent magnets. Table A1 in Appendix A presents the main characteristics of the actuator [8].

Since the cars are positioned over excited phases, a magnetic propulsion force parallel to the surface of the armature is created on the cars and pushes them accordingly, that is, the planar force of the actuator [8]. Depending on how the phases are fed with dc current in terms of value and direction, the movement of the mover can take place along the *x*- and *y*-axis directions, or any direction on a plane parallel to the armature and determined by the planar force.

## 2. Materials and Methods

The planar actuator was theoretically analyzed, designed, assembled and tested. The analytical model was proposed in order to predict the behavior of the flux density distribution and the planar force of the actuator under static conditions. Magnetic field equations were developed and computed the distribution of the magnetic flux density in the air-gap. That led to the calculation of the forces produced by the device. The analytical model represented a way to obtain the operation characteristics of the actuator based on dimensions and materials characteristics. The model was employed also as a design tool that could allow one to set the air-gap length, the dimensions of the permanent magnets, the active volume of the coils and the number of turns of each phase.

Once the main dimensions of the planar actuator were defined, a numerical model was developed to analyze the device. Its results were compared to the ones produced by the analytical model as a way to validate the latter. The numerical results were obtained by a computational package using the finite element method. The planar actuator was simulated by means of a 3D model. In the simulations, the phases located in between the cars were excited with dc current in order to calculate the forces [8]. The model generated has 315,000 elements in a volume that envelops the mover and the corresponding phases. It was divided into regions, according the materials employed in the real prototype. The four NdFeB permanent magnets were considered identical with respect of their magnetic properties. The permanent magnet regions were characterized by the relative magnetic permeability and the remanent magnetic flux density with its normal component. The back iron regions were defined as magnetically nonlinear by means of the BH curve of the SAE 1045 steel. Only two phases were represented in the model, that is, an *x*-phase and a *y*-phase, each one located between the sets of permanent magnets of the two cars, similarly to the analytical model. The model was immersed in an air region with magnetic permeability equal to *µ*_o_.

Although the numerical analysis can be a process that demands a longer time for pre-processing, particularly in 3D models, it allows the analysis of the magnetic field conditions in the ferromagnetic materials. It helps to verify the level of magnetic saturation in those regions. This is particularly significant in order to validate the analytical results since, in the analytical model, the ferromagnetic material is considered infinitely permeable.

### 2.1. Analytical Study of the Planar Actuator

**Figure 2:**
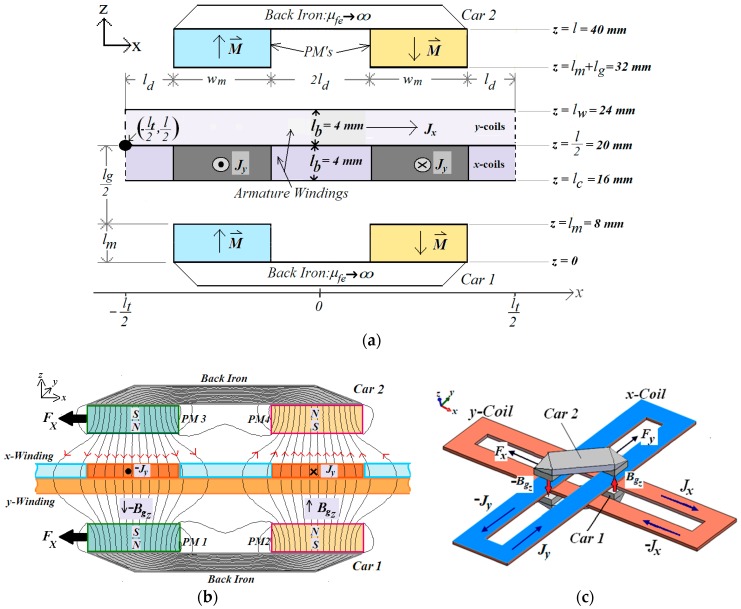
(**a**) Cut view of the model of the planar actuator for analyzing the magnetic field [8]; (**b**) cut view with the magnetic flux lines established by the PMs; (**c**) perspective view of the mover with an *x* and *y*-coils excited by current.

The planar actuator was studied using an analytical 3D model. The magnetic field produced by the permanent magnets was analyzed separately from the field produced by the winding currents. Then both fields computed in that way were added. The magnetic circuit of the planar actuator was divided into regions and boundaries where conditions for the field were imposed [8]. Figure 2a represents the magnetic circuit used by the analytical model for the study of the magnetic field and the forces when both cars were aligned. In the model, only the phases located in between the cars and subjected to the air-gap flux density produced by the permanent magnetics are considered to compute the static forces. In Figure 2b, the magnetic lines flux established through the magnetic circuit of the actuator by the PMs are presented and the directions of the current density, *z*-component of the magnetic flux density and the planar propulsion force, when only one *x*-coil is excited with current. Figure 2c shows a 3D-view of the mover with an *x*- and *y*-coils excited by dc current and the respective direction of the components of the planar propulsion force vector.

**Figure 3:**
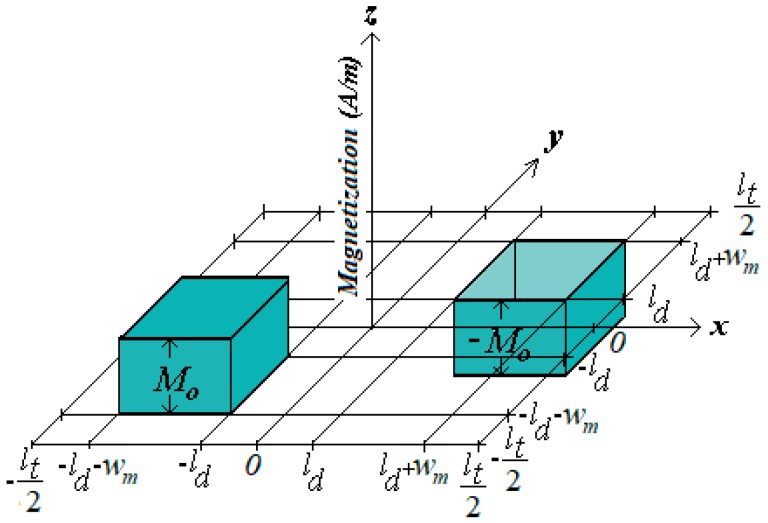
Three-dimensional characteristic of the magnetization in each pair of permanent magnets on the same car.

The spatial periodicity of the analytical model is equal to *l_t_* = 100 mm. The square area of the analytical model is ${l_{t}}^{2}$. The magnetic air-gap length is represented by *l_g_* and it is equal to the distance between the polar surfaces of the permanent magnets of Cars *1* and *2*. Both windings, *x* and *y*, are located in the air-gap. Still in Figure 2, *l_m_* is the thickness of each permanent magnet and *w_m_* is the side of the polar area of each permanent magnet; 2*l_d_* is the distance between two permanent magnets of the same car through *x* and *y* axes; *l_b_* is the thickness of each coil, *l_c_* = (*l*/2) − *l_b_*, *l_w_* = (*l*/2) + *l_b_*, $\vec{M}$ is the magnetization vector of the permanent magnets and $\vec{J}$ is the current vector density in the phase windings [8]. Figure 3 presents the three-dimensional characteristics of the magnetization in each pair of permanent magnets. The back-iron material is considered to have infinite permeability ideally, that is, $\mu_{fe}\to \infty .$ The latter is a valid approach because the magnetic flux density throughout the back iron is well below the saturation of the material due to the permanent magnet strength and the dimensions of the back iron and of the air-gap.

#### 2.1.1. Magnetic Field Produced by Permanent Magnets

In the analysis of the magnetic field produced by the permanent magnets, a formulation of the magnetic scalar potential was employed because all regions are free from current; the field produced by the current in the windings is analyzed separately. Therefore, the regions between $z=l_{c}$ and $z=l_{w}$, where the coils are located, have the same properties as the air. The magnetic field $\vec{H}$ was analyzed by representing it as the negative gradient of the magnetic scalar potential ψ so that $\vec{H}=-\nabla \psi$. Taking into account that $\nabla \cdot \vec{B}=0$ ($\vec{B}$ is the magnetic flux density) and, in the air-gap, $\vec{B}=\mu_{0}\vec{H}$, one can obtain the Laplace’s equation, in terms of the magnetic scalar potential in the air-gap $\psi_{g}$, given by $\nabla^{2}\psi_{g}=0$ [8,9].

There are two different regions that contain permanent magnets: one represents Car *1* and the other, Car *2*. In the permanent magnet regions, the relationship between vectors $\vec{B}$ and $\vec{H}$ is given by $\vec{B}=\mu_{0}\left(\vec{H}+\vec{M}\right)$ [9]. Using the same procedure as in the air-gap, the Poisson’s equation can be obtained in terms of the magnetic scalar potential in the permanent magnet regions so that $\nabla^{2}\psi_{pm}=\nabla \cdot \vec{M}$ [8,9]. In each permanent magnet region, the magnetization vector has only a *z*-component, or $\vec{M}=M_{z}\vec{k}$ and $M_{z}$ is expressed as a double Fourier series. This is a function of *x* and *y* and the periodicity is equal to *l_t_* = 8*l_d_*. Hence:

(1) $$\begin{array}{l} M_{z}=\sum_{\begin{array}{l} n=1,3,\ldots \\ m=1,3,\ldots \end{array}}^{\infty }\left[M_{nm}\sin\left(\kappa nx\right)\sin\left(\kappa my\right)\right]\\ M_{z}=-\frac{16M_{o}}{{\left(\kappa l_{t}\right)}^{2}}\sum_{\begin{array}{l} n=1,3,\ldots \\ m=1,3,\ldots \end{array}}^{\infty }\left[\begin{array}{l} \frac{\left(\cos\left(\kappa n\left(l_{d}+w_{m}\right)\right)-\cos\left(\kappa nl_{d}\right)\right)\left(\cos\left(\kappa m\left(l_{d}+w_{m}\right)\right)-\cos\left(\kappa ml_{d}\right)\right)}{nm}\\ \times \sin\left(\kappa nx\right)\sin\left(\kappa my\right) \end{array}\right] \end{array},$$

where $\kappa =2\pi /l_{t}$, $M_{o}$ is the remanent magnetization of the permanent magnets and *n* and *m* are integers. Using (1), it is possible to verify that $\nabla \cdot \vec{M}=0$, so the Poisson’s equation $\nabla^{2}\psi_{pm}=\nabla \cdot \vec{M}$ assumes the form of the Laplace’s equation, $\nabla^{2}\psi_{pm}=0$. The solution for both Laplace’s equations involves the application of boundary conditions given by [8,9,10]:

- The magnetic scalar potentials are equal to zero on the planes *x* = −*l_t_*/2, *y* = −*l_t_*/2, *x* = 0, *y* = 0, *x* = *l_t_*/2, *y* = *l_t_*/2, *z* = 0 and *z* = 2*l_m_* + *l_g_*.
- On the plane *z* = *l_m_*, the *z*-component of the magnetic flux density in the air-gap is equal to the *z*-component of the magnetic flux density in the permanent magnet region of Car *1*, $B_{g_{z}}=B_{pm1_{z}}$. On the same plane, $\psi_{g}=\psi_{pm1}$.
- On the plane *z* = *l_m_* + *l_g_*, the *z*-component of the magnetic flux density in the air-gap is equal to the *z*-component of the magnetic flux density in the permanent magnet region of Car *2*, $B_{g_{z}}=B_{pm2_{z}}$. On the same plane, $\psi_{g}=\psi_{pm2}$.

By means of those conditions, the solution of the potential equations can be obtained [5]. The equations for the *x*, *y* and *z*-components of the magnetic flux density vector in the air-gap are obtained from $-\mu_{o}\partial \psi_{g}/\partial x$, $-\mu_{o}\partial \psi_{g}/\partial y$ and $-\mu_{o}\partial \psi_{g}/\partial z$. They are given by [8]:

(2) $$B_{g_{x}}=\frac{8\mu_{0}M_{o}}{\kappa {l_{t}}^{2}}\sum_{\begin{array}{l} n=1,3,\ldots \\ m=1,3,\ldots \end{array}}^{\infty }\left[\begin{array}{l} \frac{\left(\cos\left(\kappa n\left(l_{d}+w_{m}\right)\right)-\cos\left(\kappa nl_{d}\right)\right)\left(\cos\left(\kappa m\left(l_{d}+w_{m}\right)\right)-\cos\left(\kappa ml_{d}\right)\right)}{\gamma m}\times \\ \left(\frac{k_{3}e^{\gamma z}+k_{4}e^{-\gamma z}}{1-e^{-\gamma (4l_{m}+2l_{g})}}\right)\cos\left(\kappa nx\right)\sin\left(\kappa my\right) \end{array}\right],$$

(3) $$B_{g_{y}}=\frac{8\mu_{0}M_{o}}{\kappa {l_{t}}^{2}}\sum_{\begin{array}{l} n=1,3,\ldots \\ m=1,3,\ldots \end{array}}^{\infty }\left[\begin{array}{l} \frac{\left(\cos\left(\kappa n\left(l_{d}+w_{m}\right)\right)-\cos\left(\kappa nl_{d}\right)\right)\left(\cos\left(\kappa m\left(l_{d}+w_{m}\right)\right)-\cos\left(\kappa ml_{d}\right)\right)}{\gamma n}\times \\ \left(\frac{k_{3}e^{\gamma z}+k_{4}e^{-\gamma z}}{1-e^{-\gamma (4l_{m}+2l_{g})}}\right)\sin\left(\kappa nx\right)\cos\left(\kappa my\right) \end{array}\right],$$

(4) $$B_{g_{z}}=\frac{8\mu_{0}M_{o}}{{\left(\kappa l_{t}\right)}^{2}}\sum_{\begin{array}{l} n=1,3,\ldots \\ m=1,3,\ldots \end{array}}^{\infty }\left[\begin{array}{l} \frac{\left(\cos\left(\kappa n\left(l_{d}+w_{m}\right)\right)-\cos\left(\kappa nl_{d}\right)\right)\left(\cos\left(\kappa m\left(l_{d}+w_{m}\right)\right)-\cos\left(\kappa ml_{d}\right)\right)}{nm}\times \\ \left(\frac{k_{3}e^{\gamma z}-k_{4}e^{-\gamma z}}{1-e^{-\gamma (4l_{m}+2l_{g})}}\right)\sin\left(\kappa nx\right)\sin\left(\kappa my\right) \end{array}\right],$$

where $\mu_{0}$ is the magnetic permeability of the vacuum, $\gamma =\kappa \sqrt{n^{2}+m^{2}}$ and *k*_3_ and *k*_4_ are given by [8]:

(5) $$k_{3}=e^{-\gamma (5l_{m}+2l_{g})}-e^{-\gamma (l_{m}+l_{g})}-e^{-\gamma (3l_{m}+2l_{g})}+e^{-\gamma (3l_{m}+l_{g})},$$

(6) $$k_{4}=e^{\gamma l_{m}}-e^{-\gamma l_{m}}-e^{-\gamma (3l_{m}+l_{g})}+e^{-\gamma (l_{m}+l_{g})}.$$

#### 2.1.2. The Equation of the Planar Propulsion Force

The propulsion force that acts on the mover and produces movement over the plane was obtained by means of the Laplace’s force equation $\vec{F}=\int_{V}\left(\vec{J}\times \vec{B}\right)\;dv$ [2,3,4,5]. This equation can be applied to the analysis of the planar actuator [8]. This assumes that the resulting force vector on the mover is the result of the interaction between the *z*-component of the magnetic flux density vector in the air-gap, $B_{g_{z}}$ and the current density vector in the *x*-phases, ${\vec{J}}_{Xw}=J_{y}\vec{j}$ and between $B_{g_{z}}$ and the current density vector in the *y*-phases, ${\vec{J}}_{Yw}=J_{x}\vec{i}$. Therefore:

(7) $$\begin{array}{l} {\vec{F}}_{P}=-2\left[\left(\int_{l_{c}}^{\frac{l}{2}}\int_{0}^{\frac{l_{t}}{2}}\int_{l_{d}}^{\frac{l_{t}}{2}-l_{d}}\left(J_{o_{y}}\vec{j}\times B_{g_{z}}\vec{k}\right)dv\right)+\left(\int_{\frac{l}{2}}^{l_{w}}\int_{l_{d}}^{\frac{l_{t}}{2}-l_{d}}\int_{0}^{\frac{l_{t}}{2}}\left(J_{o_{x}}\vec{i}\times B_{g_{z}}\vec{k}\right)dv\right)\right]\\ {\vec{F}}_{P}=2\left[\left(-\int_{l_{c}}^{\frac{l}{2}}\int_{0}^{\frac{l_{t}}{2}}\int_{l_{d}}^{\frac{l_{t}}{2}-l_{d}}\left(J_{o_{y}}B_{g_{z}}\right)dv\right)\vec{i}+\left(\int_{\frac{l}{2}}^{l_{w}}\int_{l_{d}}^{\frac{l_{t}}{2}-l_{d}}\int_{0}^{\frac{l_{t}}{2}}\left(J_{o_{x}}B_{g_{z}}\right)dv\right)\vec{j}\right]\\ {\vec{F}}_{P}=\sum_{\begin{array}{l} n=1,3,\ldots \\ m=1,3,\ldots \end{array}}^{\infty }\left[\begin{array}{l} \frac{32\mu_{0}l_{t}M_{o}\cos\left(\kappa nl_{d}\right)\cos\left(\kappa ml_{d}\right)}{\pi^{3}\kappa \gamma {\left(nm\right)}^{2}\left(1-e^{-\gamma (4l_{m}+2l_{g})}\right)}\times \\ \left[\begin{array}{l} {}[-J_{o_{y}}\cos\left(\kappa nl_{d}\right)[k_{3}(e^{\gamma l/2}-e^{\gamma ((l/2)-l_{g})})+k_{4}(e^{-\gamma l/2}-e^{-\gamma ((l/2)-l_{g})})]\vec{i}]\\ +[J_{o_{x}}\cos\left(\kappa ml_{d}\right)[k_{3}(e^{\gamma ((l/2)+l_{g})}-e^{\gamma l/2})+k_{4}(e^{-\gamma ((l/2)+l_{g})}-e^{-\gamma l/2})]\vec{j} \end{array}\right] \end{array}\right] \end{array},$$

where $J_{oy}$ is the current density in one phase of the *x*-winding and $J_{ox}$, the current density in one phase of the *y*-winding. When fed by current, the *x*-phases produce a propulsion force through the direction of the *x*-axis and the *y*-phases, through the direction of the *y*-axis. In Equation (7), it is assumed that only one phase of the *x*-winding and of *y*-winding are fed by current and both are aligned with the permanent magnets of the mover.

#### 2.1.3. Magnetic Field Produced by Current in the Phases

The magnetic flux density vector produced by the winding current was obtained by employing the formulation of the magnetic vector potential [11]. The magnetic field produced by *x*-coils were analyzed separately of the magnetic field produced by *y*-coils. Therefore, in the model of Figure 2, there are two regions free from currents: between *z* = 0 and *z* = *l_c_* and between *z* = *l*/2 and *z* = *l*, when only the effect of the current in *x*-coils is taken in account. The effect of the magnetization of the permanent magnets is not considered because the magnetic field produced by them was analyzed separately in Section 2.1.1.

In the formulation of the vector magnetic potential, the magnetic flux density vector, $\vec{B}$, can be expressed as the curl of $\vec{A}$, or $\vec{B}=\nabla \times \vec{A}$ [4]. The curl of the magnetic field vector, $\vec{H}$, is equal to the current density vector, that is, $\nabla \times \vec{H}=\vec{J}$. Consequently, the curl of $\vec{B}$ results in $\nabla \times \vec{B}=\nabla \times \nabla \times \vec{A}=\mu \vec{J}$. The magnetic vector potential is obtained by means of the current density vector. In an *x*-coil phase, the current density vector is expressed by a Fourier series given by:

(8) $${\vec{J}}_{Xw}=J_{y}\vec{j}=\frac{4J_{oy}}{\pi }\sum_{n=1,3,\ldots }^{\infty }\left(\frac{\cos\left(\kappa nl_{d}\right)}{n}\sin\left(\kappa nx\right)\right)\vec{j}.$$

The magnetic field produced by an *x*-coil phase with current was calculated separately from the magnetic field produced by a *y*-coil phase. In an *x*-winding phase, $\nabla \times \nabla \times {\vec{A}}_{Xw}=\mu_{0}J_{y}\vec{j}$, where ${\vec{A}}_{Xw}$ is the magnetic vector potential in the *x*-winding region. After some manipulation, the equation $\nabla^{2}A_{Xw_{y}}=-\mu_{0}J_{y}$ is obtained, where $A_{Xw_{y}}$ is the *y*-component of $A_{Xw}$. This scalar equation is the Poisson’s equation in terms of the vector magnetic potential ${\vec{A}}_{Xw}$ [5]. Its solution is the result of the sum of two terms: a homogeneous term that must satisfy the Laplace’s equation and a particular term solved by the Poisson’s equation. In the regions with no current, the curl of $\vec{B}$ assumes the form of Laplace’s equation, $\nabla^{2}A_{g1_{y}}=0$ and $\nabla^{2}A_{g2_{y}}=0$, where $A_{g1_{y}}$ and $A_{g2_{y}}$ are the *y*-components of magnetic vector potentials in the current-free space regions related to the current density in the *x*-phase. The magnetic vector potential in the region between *z* = 0 and *z* = *l_c_* (air-gap 1) is represented by $A_{g1_{y}}$ and between *z* = *l*/2 and *z* = *l* (air-gap 2), by $A_{g2_{y}}$. The solution for both Laplace’s equation and Poisson’s equation involves the application of boundary conditions given by [10,11]:

- The magnetic vector potentials are equal to zero on the planes *x* = −*l_t_*/2, *y* = −*l_t_*/2, *x* = 0, *y* = 0, *x* = *l_t_*/2, *y* = *l_t_*/2.
- On the plane *z* = 0, the tangential component of the magnetic flux density vector in the air-gap 1, $B_{g1_{x}}$, obtained from $-\partial A_{g1_{y}}/\partial z$, is equal to zero.
- On the plane *z* = 2*l_m_* + *l_g_*, the tangential component of the magnetic flux density vector in the air-gap 2, $B_{g2_{x}}$, is equal to zero.
- On the plane *z* = *l_c_*, the *z*-component of the magnetic flux density in the air-gap 1 is equal to the *z*-component of the magnetic flux density in the *x*-coil region, $B_{g1_{z}}=B_{Xw\;z}$. On the same plane, the *x*-components of the magnetic field vector in the air-gap 1 and in the *x*-coil region are also equal so that $H_{g1_{x}}=H_{Xwx}$.
- On the plane *z* = *l*/2, the *z*-component of the magnetic flux density in the *x*-coil region is equal to the *z*-component of the magnetic flux density in the air-gap 2, $B_{g2_{z}}=B_{Xw\;z}$. On the same plane, the *x*-components of the magnetic field vector in the *x*-coil region and in the air-gap 2 are again equal so that $H_{g2_{x}}=H_{Xwx}$.

These conditions allow the solution of the potential equations. The equations for *x* and *z*-components of the magnetic flux density vector in the air-gap 1, $B_{g1_{x}}$ and $B_{g1_{z}}$, produced by the current in the *x*-coil phase, are obtained from $-\partial A_{g1_{y}}/\partial z$ and $\partial A_{g1_{y}}/\partial x$.

For the *y*-winding phases, the current vector is represented by a Fourier series given by:

(9) $${\vec{J}}_{Yw}=J_{x}\vec{i}=\frac{4J_{ox}}{\pi }\sum_{m=1,3,\ldots }^{\infty }\left(\frac{\cos\left(\kappa ml_{d}\right)}{m}\sin\left(\kappa mx\right)\right)\vec{i},$$

and $\nabla \times \nabla \times {\vec{A}}_{Yw}=\mu_{0}J_{x}\vec{i}$, where ${\vec{A}}_{Yw}$ is the magnetic vector potential in the *y*-winding region and its *x*-component is represented by $A_{Yw_{x}}$. Again, after some manipulation, $\nabla^{2}A_{Yw_{x}}=-\mu_{0}J_{x}$ is obtained and its solution is the result of the sum of two terms: a homogeneous term that must satisfy the Laplace’s equation and a particular term solved by the Poisson’s equation. In the regions with no current, $\nabla^{2}A_{a1_{x}}=0$ and $\nabla^{2}A_{a2_{x}}=0$, where $A_{a1_{x}}$ and $A_{a2_{x}}$ are the *x*-components of magnetic vector potentials in the current-free space regions limited by the planes *z* = 0 and *z* = *l*/2 (air-gap 1) and by *z* = *l_w_* and *z* = *l* (air-gap 2). Both regions are related to the current in the *y*-winding. Analogous boundary conditions are used on the planes that limit the regions. They allow solution of the potential equations. The equations for *y* and *z*-components of the magnetic flux density vector in air-gap 1, $B_{a1_{y}}$ and $B_{a1_{z}}$, produced by the current in the *y*-coil phase, are obtained from $\partial A_{a1_{x}}/\partial z$ and $-\partial A_{a1_{x}}/\partial y$.

When the *x* and *y*-phases located between the permanent magnets of the cars are fed by current, the resulting *x*, *y* and *z*-components magnetic flux density in air-gap 1 are given by [8]:

(10) $$B_{g1_{x}}=-\frac{2\mu_{0}J_{o_{y}}}{\kappa \pi }\sum_{n=1,3,\ldots }^{\infty }\left[A_{1}\frac{\cos\left(\kappa nl_{d}\right)}{n^{2}}\left(e^{\kappa nz}-e^{-\kappa nz}\right)\sin\left(\kappa nx\right)\right],$$

(11) $$B_{a1_{y}}=\frac{2\mu_{0}J_{o}x}{\kappa \pi }\sum_{m=1,3,\ldots }^{\infty }\left[B_{1}\frac{\cos\left(\kappa ml_{d}\right)}{m^{2}}\left(e^{\kappa mz}-e^{-\kappa mz}\right)\sin\left(\kappa my\right)\right],$$

(12) $$B_{g1_{z}}+B_{a1_{z}}=\frac{2\mu_{0}}{\kappa \pi }\left[\begin{array}{l} \sum_{n=1,3,\ldots }^{\infty }\left[A_{1}J_{o_{y}}\frac{\cos\left(\kappa nl_{d}\right)}{n^{2}}\left(e^{\kappa nz}+e^{-\kappa nz}\right)\cos\left(\kappa nx\right)\right]+\\ -\sum_{m=1,3,\&}^{\infty }\left[B_{1}J_{o_{x}}\frac{\cos\left(\kappa ml_{d}\right)}{m^{2}}\left(e^{\kappa mz}+e^{-\kappa mz}\right)\cos\left(\kappa my\right)\right] \end{array}\right],$$

where $A_{1}$ and $B_{1}$ are given by [8]:

(13) $$A_{1}=\left[\left(\frac{1}{e^{\kappa nl_{c}}-e^{-\kappa nl_{c}}-e^{\kappa n\left(2l+l_{c}\right)}+e^{\kappa n\left(2l-l_{c}\right)}}\right)\left(\begin{array}{l} e^{2\kappa nl_{c}}-e^{2\kappa nl}+e^{2\kappa n(l-l_{c})}-1-e^{\kappa n\left(l/2+l_{c}\right)}+\\ +e^{\kappa n\left(l/2-l_{c}\right)}+e^{\kappa n\left(\left(3l/2\right)+l_{c}\right)}-e^{\kappa n\left(\left(3l/2\right)-l_{c}\right)} \end{array}\right)\right],$$

(14) $$B_{1}=\left[\left(\frac{1}{e^{\kappa ml/2}-e^{-\kappa ml/2}-e^{5\kappa ml/2}+e^{3\kappa ml/2}}\right)\left(\begin{array}{l} 2e^{\kappa ml}-1-e^{\kappa m\left(l/2+l_{w}\right)}+e^{-\kappa m\left(l/2-l_{w}\right)}-e^{2\kappa ml}+\\ -e^{\kappa m\left(3l/2-l_{w}\right)}+e^{\kappa m\left(5l/2-l_{w}\right)} \end{array}\right)\right].$$

The total magnetic flux density vector in air-gap 1, ${\vec{B}}_{T1}$, is the vector sum of the magnetic flux density vectors produced by the permanent magnets and by the current through the *x* and *y*-phases. Its components are obtained from $B_{T1_{x}}=B_{g_{x}}+B_{g1_{x}}$, $B_{T1_{y}}=B_{g_{y}}+B_{a1_{y}}$ and $B_{T1_{z}}=B_{g_{z}}+B_{g1_{z}}+B_{a1_{z}}$.

#### 2.1.4. The Equation of the Normal Force

In the planar actuator, a normal attraction force is produced between Cars *1* and *2*. This is mainly due to the magnetic field produced by the permanent magnets. The magnetic field produced by the phase currents is smaller when compared to the permanent magnet field. Therefore, its effects on the production of the normal force are not significant. The effect of the normal force produced by the permanent magnet field is the magnetic attraction between Cars *1* and *2* and that keeps them magnetically coupled. In the analysis of the normal force, a Maxwell Stress Tensor, $\vec{T}$, was used [12]. The vector force is related to the Maxwell tensor by $\vec{F}=\frac{1}{\mu }\oint_{S}\vec{T}dS$. In order to calculate the normal attraction force between the cars, a closed surface of integration is used for Car *1*. The lower surface is located on *y* = −∞, where the magnetic field is considered equal to zero and, on the lateral sides, the integration over the terms of the tensor related to these sides cancel each other [5]. On the upper surface, located on the plane *z* = *l_m_*, the integration is made over the tensor term $T_{zz}={B_{z}}^{2}-\frac{1}{2}{\left|B\right|}^{2}$. In this way, the normal force on the Car *1* is calculated from [8]:

(15) $$\begin{array}{l} {\vec{F}}_{N}=\frac{2}{2\mu_{o}}\int_{0}^{\frac{l_{t}}{2}}\int_{0}^{\frac{l_{t}}{2}}{\langle \left({B_{T1z}}^{2}-{B_{T1x}}^{2}-{B_{T1y}}^{2}\right)\rangle }_{xy}dxdy\vec{k}\\ {\vec{F}}_{N}=\frac{4}{{l_{t}}^{2}}\mu_{o}\{\begin{array}{l} \sum_{\begin{array}{l} n=1,3,\ldots \\ m=1,3,\ldots \end{array}}^{\infty }[\begin{array}{l} {\left[\frac{M_{o}(\cos\left(\kappa n(l_{d}+w_{m})\right)-\cos\left(\kappa nl_{d}\right))\cdot (\cos\left(\kappa m(l_{d}+w_{m})\right)-\cos\left(\kappa ml_{d}\right))}{1-e^{-\gamma (4l_{m}+2l_{g})}}\right]}^{2}\times \\ \left[\frac{\gamma^{2}{\left(k_{3}e^{\gamma lm}-k_{4}e^{-\gamma lm}\right)}^{2}-\kappa^{2}(n^{2}+m^{2}){\left(k_{3}e^{\gamma lm}+k_{4}e^{-\gamma lm}\right)}^{2}}{{\left(\kappa^{2}\gamma nm\right)}^{2}}\right] \end{array}]+\\ \left[\frac{{l_{t}}^{4}}{2{\left(\kappa \pi \right)}^{2}}[\begin{array}{l} \sum_{n=1,3,\ldots }^{\infty }{\left(\frac{A_{1}J_{o_{y}}\cos\left(\kappa nl_{d}\right)}{n^{2}}\right)}^{2}+\\ \sum_{m=1,3,\ldots }^{\infty }{\left(\frac{B_{1}J_{o_{x}}\cos\left(\kappa ml_{d}\right)}{m^{2}}\right)}^{2} \end{array}]\right] \end{array}\}\vec{k} \end{array},$$

where the subscript *T1* in $\left({B_{T1z}}^{2}-{B_{T1x}}^{2}-{B_{T1y}}^{2}\right)$ indicates total values of the components of the magnetic flux density vector that results of the vector sum of the magnetic flux density vectors produced in air-gap 1 by the permanent magnets and by the current in the *x* and *y*-phases.

#### 2.1.5. Decoupling Forces

The stall force is the minimum force necessary to break the magnetic coupling between the upper and the lower cars. When one car is subjected to an external force through the *x*- or *y*-axis, the cars can become out of alignment with respect to each the other and the planar actuator cannot work properly. In order to analyze the decoupling force, a set of equations was developed to calculate the values of the planar force produced between the cars when one of them suffers a displacement with relation to the other. An external force applied to one car can break the magnetic coupling when it is higher than the attraction forces that tend to keep the cars magnetically coupled [8].

**Figure 4:**
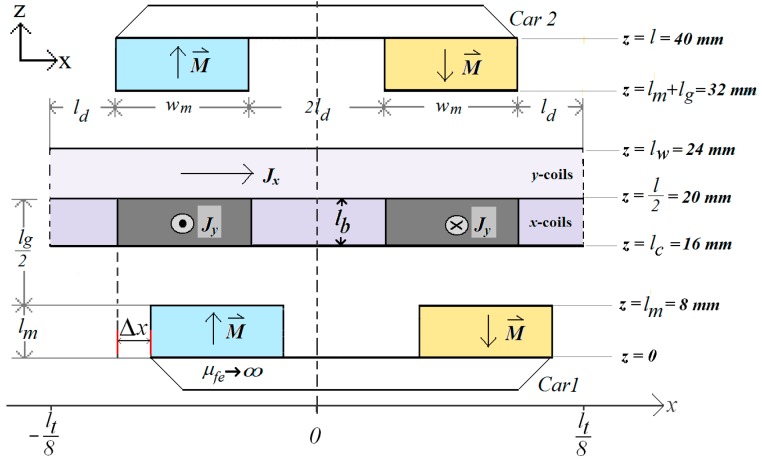
Cut view of the model of the planar actuator for analyzing the magnetic field when there is a displacement Δ*x* between the cars.

In the decoupling model, the spatial periodicity, *l_t_*, shown in the model represented by Figure 2, is increased to become higher than 100 mm, resulting in 400 mm, in order to allow the analysis of the displacement of one car with respect to the other, according to Figure 4. The equations produce the values for the attractive planar force between the cars when there are displacements Δ*x* and Δ*y* between them, where Δ*x* is the displacement through the *x*-axis and Δ*y* through the *y*-axis [8]. In the analytical model, the position of the Car *2* is kept at the same coordinates while the displacement is applied to the Car *1*.

The magnetization expression of Car *2* is given by (1) and Car *1* is represented by:

(16) $$M_{1z}=-\frac{16M_{o}}{{\left(\kappa l_{t}\right)}^{2}}\sum_{\begin{array}{l} n=1,3,\ldots \\ m=1,3,\ldots \end{array}}^{\infty }\left[\begin{array}{l} \frac{\left(\cos\left(\kappa n\left(l_{d}+w_{m}\right)\right)-\cos\left(\kappa nl_{d}\right)\right)\cdot \left(\cos\left(\kappa m\left(l_{d}+w_{m}\right)\right)-\cos\left(\kappa ml_{d}\right)\right)}{nm}\\ \times \sin\left(\kappa n\left(x\pm \Delta x\right)\right)\sin\left(\kappa m\left(y\pm \Delta y\right)\right) \end{array}\right].$$

Following the same steps presented in Section 2.1.1, the equations for the components of the magnetic flux density vector produced by the permanent magnets as a function of the displacement of Car *1* with respect to Car *2* can be obtained. For the air-gap, those components are [8]:

(17) $$B_{g_{x}}=-\frac{8\mu_{0}M_{o}}{\kappa {l_{t}}^{2}}\sum_{\begin{array}{l} n=1,2,\ldots \\ m=1,2,\ldots \end{array}}^{\infty }\left[\frac{M_{1}}{m\gamma \left(1-e^{-\gamma \left(4l_{m}+2l_{g}\right)}\right)}\cdot \left(\left(S_{x1}C_{1}+S_{x2}C_{2}\right)e^{\gamma z}+\left(S_{x1}C_{3}-S_{x2}C_{2}\right)e^{-\gamma z}\right)\right],$$

(18) $$B_{g_{y}}=-\frac{8\mu_{0}M_{o}}{\kappa {l_{t}}^{2}}\sum_{\begin{array}{l} n=1,2,\ldots \\ m=1,2,\ldots \end{array}}^{\infty }\left[\frac{M_{1}}{n\gamma \left(1-e^{-\gamma \left(4l_{m}+2l_{g}\right)}\right)}\cdot \left(\left(S_{y1}C_{1}+S_{y2}C_{2}\right)e^{\gamma z}+\left(S_{y1}C_{3}-S_{y2}C_{2}\right)e^{-\gamma z}\right)\right],$$

(19) $$B_{g_{z}}=-\frac{8\mu_{0}M_{o}}{\kappa^{2}{l_{t}}^{2}}\sum_{\begin{array}{l} n=1,2,\ldots \\ m=1,2,\ldots \end{array}}^{\infty }\left[\frac{M_{1}}{nm\left(1-e^{-\gamma \left(4l_{m}+2l_{g}\right)}\right)}\cdot \left(\left(S_{z1}C_{1}+S_{z2}C_{2}\right)e^{\gamma z}-\left(S_{z1}C_{3}-S_{z2}C_{2}\right)e^{-\gamma z}\right)\right],$$

where $C_{1}=e^{-\gamma \left(5l_{m}+2l_{g}\right)}-e^{-\gamma \left(3l_{m}+2l_{g}\right)}$, $C_{2}=e^{-\gamma \left(3l_{m}+l_{g}\right)}-e^{-\gamma \left(l_{m}+l_{g}\right)}$ and $C_{3}=e^{\gamma l_{m}}-e^{-\gamma l_{m}}$. $M_{1}$, *S_x_*_1_, *S_x_*_2_, *S_y_*_1_, *S_y_*_2_, *S_z_*_1_ and *S_z_*_2_ are given by:

(20) $$M_{1}=-\left[\left(\cos\left(\kappa n\left(l_{d}+w_{m}\right)\right)-\left(\cos\left(\kappa nl_{d}\right)\right)\right)\times \left(\cos\left(\kappa m\left(l_{d}+w_{m}\right)\right)-\left(\cos\left(\kappa ml_{d}\right)\right)\right)\right],$$

(21) $$S_{x1}=\cos\left(\kappa n\left(x\pm \Delta x\right)\right)\sin\left(\kappa m\left(y\pm \Delta y\right)\right),\;S_{x2}=\cos\left(\kappa nx\right)\sin\left(\kappa my\right),$$

(22) $$S_{y1}=\sin\left(\kappa n\left(x\pm \Delta x\right)\right)\cos\left(\kappa m\left(y\pm \Delta y\right)\right),\;S_{y2}=\sin\left(\kappa nx\right)\cos\left(\kappa my\right),$$

(23) $$S_{z1}=\sin\left(\kappa n\left(x\pm \Delta x\right)\right)\sin\left(\kappa m\left(y\pm \Delta y\right)\right),\;S_{z2}=\sin\left(\kappa nx\right)\sin\left(\kappa my\right).$$

When the cars are misaligned with respect to each other, the attractive planar force tends to realign them and it can act through the *x* or *y*-axis or any other direction on the plane, if the displacement occurs in the *x* or *y* coordinates or in both. The equations for the attractive planar force ${\vec{F}}_{d_{p}}$ that tend to keep the cars magnetically coupled were obtained by means of the Maxwell Stress Tensor [8,9]. The planar force has *x* and *y*-components and can be computed from [8]:

(24) $$\begin{array}{l} {\vec{F}}_{d_{p}}=F_{d_{p}x}\vec{i}+F_{d_{p_{y}}}\vec{j}\\ {\vec{F}}_{d_{p}}=\frac{1}{2\mu_{o}}\left[\int_{0}^{\frac{l_{t}}{2}}\int_{0}^{l_{t}}{\langle B_{g_{x}}B_{g_{z}}\rangle }_{xy}dx\;dy\;\vec{i}+\int_{0}^{\frac{l_{t}}{2}}\int_{0}^{l_{t}}{\langle B_{g_{y}}B_{g_{z}}\rangle }_{xy}dxdy\vec{j}\right] \end{array}$$

(25) $$F_{d_{p}x}=\frac{l_{t}^{2}}{8\mu_{0}}\sum_{\begin{array}{l} n=1,2,..\\ m=1,2,. \end{array}}^{\infty }A_{1}A_{3}\left[\left(B_{1}B_{4}-B_{2}B_{3}\right)\sin\left(\kappa n\left(\pm \Delta x\right)\right)\cos\left(\kappa m\left(\pm \Delta y\right)\right)\right],$$

(26) $$F_{d_{p_{y}}}=\frac{l_{t}^{2}}{8\mu_{0}}\sum_{\begin{array}{l} n=1,2,..\\ m=1,2,. \end{array}}^{\infty }A_{2}A_{3}\left[\left(B_{1}B_{4}-B_{2}B_{3}\right)\cos\left(\kappa n\left(\pm \Delta x\right)\right)\sin\left(\kappa m\left(\pm \Delta y\right)\right)\right],$$

where $A_{1}=-8\frac{\mu_{o}M_{o}M_{1}}{a{l_{t}}^{2}\kappa \gamma m}$, $A_{2}=-8\frac{\mu_{o}M_{o}M_{1}}{a{l_{t}}^{2}\kappa \gamma n}$, $A_{3}=-8\frac{\mu_{o}M_{o}M_{1}}{a{\left(l_{t}\kappa \right)}^{2}nm}$, $a=1-e^{-\gamma \left(4l_{m}+2l_{g}\right)}$

$B_{1}=C_{1}e^{\gamma l_{m}}+C_{3}e^{-\gamma l_{m}}$, $B_{2}=C_{2}\left(e^{\gamma l_{m}}-e^{-\gamma l_{m}}\right)$, $B_{3}=C_{1}e^{\gamma l_{m}}-C_{3}e^{-\gamma l_{m}}$ and $B_{4}=C_{2}\left(e^{\gamma l_{m}}+e^{-\gamma l_{m}}\right)$
$C_{1}=e^{-\gamma \left(5l_{m}+2l_{g}\right)}-e^{-\gamma \left(3l_{m}+2l_{g}\right)}$, $C_{2}=e^{-\gamma \left(3l_{m}+l_{g}\right)}-e^{-\gamma \left(l_{m}+l_{g}\right)}$ and $C_{3}=e^{\gamma l_{m}}-e^{-\gamma l_{m}}$ [8].

#### 2.1.6. Displacement between the Mover and an Excited Phase

**Figure 5:**
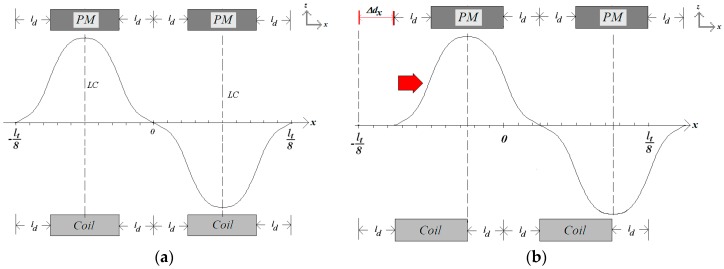
(**a**) Excited coil of the *x*-winding centralized with relation to the magnetic flux density produced by the PM’s; (**b**) displacement $\Delta d_{x}$ between the PM’s and the excited coil.

In Figure 5a, an excited phase of the *x*-winding is aligned with relation to the magnetic flux density produced by the PM’s. In this situation, the *x*-component of the propulsion force vector presents the higher value for a given current. When there is a displacement between the PMs and an excited phase, as shown in Figure 5b, the propulsion force is smaller since the displacement is force. Ideally, when the displacement is higher than 2*l_d_*, the propulsion force will be produced in the inverse direction in relation to that produced when the displacement assumes values between 0 and 2*l_d_*. Consequently, if the mover must develop a continuous movement in one direction, coils must be excited sequentially, according to the position and speed of the mover, to produce movement and avoid the inversion of the direction of the force.

In Figure 5b, the magnetic flux density of the PMs has a displacement $\Delta d_{x}$ along the *x*-axis with a relationship to the excited coil. Taking into account displacements along the *x*- and the *y*-axis, $\Delta d_{x}$ and $\Delta d_{y}$, the equation of the planar propulsion force, ${\vec{F}}_{Pd}$, produced on the mover can be obtained from the Laplace’s force and is computed from:

(27) $$\begin{array}{l} {\vec{F}}_{Pd}=-2\left[\left(\int_{l_{c}}^{\frac{l}{2}}\int_{0}^{\frac{l_{t}}{8}}\int_{l_{d}}^{l_{d}+w_{m}}\left(J_{o_{y}}\vec{j}\times B_{g_{z}}\vec{k}\right)dv\right)+\left(\int_{\frac{l}{2}}^{l_{w}}\int_{l_{d}}^{l_{d}+w_{m}}\int_{0}^{\frac{l_{t}}{8}}\left(J_{o_{x}}\vec{i}\times B_{g_{z}}\vec{k}\right)dv\right)\right]\\ {\vec{F}}_{Pd}=\sum_{\begin{array}{l} n=1,3,\ldots \\ m=1,3,\ldots \end{array}}^{\infty }[\begin{array}{l} \frac{16\mu_{0}M_{o}M_{1}}{\kappa^{4}{\left(nml_{t}\right)}^{2}\gamma a}\times \\ \left[\begin{array}{l} {}[\begin{array}{l} -J_{o_{y}}\left[\cos\left(\kappa n\left(l_{d}+w_{m}-\Delta d_{x}\right)\right)-\cos\left(\kappa n\left(l_{d}-\Delta d_{x}\right)\right)\right]\left[\cos\left(\kappa m\left(l_{t}/8\right)\right)-1\right]\times \\ \left[k_{3}\left(e^{\gamma l/2}-e^{\gamma \left(\left(l/2\right)-l_{b}\right)}\right)+k_{4}\left(e^{-\gamma l/2}-e^{-\gamma \left(\left(l/2\right)-l_{b}\right)}\right)\right] \end{array}]\vec{i}\\ +[\begin{array}{l} J_{o_{x}}\left[\cos\left(\kappa n\left(l_{t}/8\right)\right)-1\right]\left[\cos\left(\kappa m\left(l_{d}+w_{m}-\Delta d_{y}\right)\right)-\cos\left(\kappa m\left(l_{d}-\Delta d_{y}\right)\right)\right]\times \\ \left[k_{3}\left(e^{\gamma \left(\left(l/2\right)+l_{b}\right)}-e^{\gamma l/2}\right)+k_{4}\left(e^{-\gamma \left(\left(l/2\right)+l_{b}\right)}-e^{-\gamma l/2}\right)\right] \end{array}]\vec{j} \end{array}\right] \end{array}] \end{array}.$$

When there is a displacement between the mover and an excited phase, a normal force is also present due to the interaction between the current and the magnetic field produced by PMs. The effect of this force is quite different of that one analyzed in Section 2.1.4. The direction of normal force that acts on the mover depends on the direction of the current in the excited phase. It can act on the mover in the positive or negative direction of *z*-axis. When the displacement between the mover and an excited phase is zero, this kind of normal force is ideally zero. This is the reason why in Section 2.1.2, only the propulsion force was presented. The normal force is obtained from:

(28) $$\begin{array}{l} {\vec{F}}_{Pd}=-2\left[\left(\int_{l_{c}}^{\frac{l}{2}}\int_{0}^{\frac{l_{t}}{8}}\int_{l_{d}}^{l_{d}+w_{m}}\left(J_{o_{y}}\vec{j}\times B_{g_{x}}\vec{i}\right)dv\right)+\left(\int_{\frac{l}{2}}^{l_{w}}\int_{l_{d}}^{l_{d}+w_{m}}\int_{0}^{\frac{l_{t}}{8}}\left(J_{o_{x}}\vec{i}\times B_{g_{y}}\vec{j}\right)dv\right)\right]\\ {\vec{F}}_{Nd}=2\left[\left(\int_{l_{c}}^{\frac{l}{2}}\int_{0}^{\frac{l_{t}}{8}}\int_{l_{d}}^{l_{d}+w_{m}}\left(J_{o_{y}}B_{g_{x}}\right)dv\right)\vec{k}-\left(\int_{\frac{l}{2}}^{l_{w}}\int_{0}^{\frac{l_{t}}{2}}\int_{l_{d}}^{\frac{l_{t}}{2}-l_{d}}\left(J_{o_{x}}B_{g_{y}}\right)dv\right)\vec{k}\right]\\ {\vec{F}}_{Nd}=\sum_{\begin{array}{l} n=1,3,\ldots \\ m=1,3,\ldots \end{array}}^{\infty }[\begin{array}{l} \frac{16\mu_{0}M_{o}M_{1}}{\kappa^{3}{\left(\gamma ml_{t}\right)}^{2}na}\times \\ \left[\begin{array}{l} {}[\begin{array}{l} J_{o_{y}}\left[\sin\left(\kappa n\left(l_{d}+w_{m}+\Delta d_{x}\right)\right)-\sin\left(\kappa n\left(l_{d}+\Delta d_{x}\right)\right)\right]\left[\cos\left(\kappa m\left(l_{t}/8\right)\right)-1\right]\times \\ \left[k_{3}\left(e^{\gamma l/2}-e^{\gamma \left(\left(l/2\right)-l_{b}\right)}\right)-k_{4}\left(e^{-\gamma l/2}-e^{-\gamma \left(\left(l/2\right)-l_{b}\right)}\right)\right] \end{array}]\vec{k}\\ +[\begin{array}{l} -J_{o_{x}}\left[\cos\left(\kappa n\left(l_{t}/8\right)\right)-1\right]\left[\sin\left(\kappa m\left(l_{d}+w_{m}+\Delta d_{y}\right)\right)-\sin\left(\kappa m\left(l_{d}+\Delta d_{y}\right)\right)\right]\times \\ \left[k_{3}\left(e^{\gamma \left(\left(l/2\right)+l_{b}\right)}-e^{\gamma l/2}\right)-k_{4}\left(e^{-\gamma \left(\left(l/2\right)+l_{b}\right)}-e^{-\gamma l/2}\right)\right] \end{array}]\vec{k} \end{array}\right] \end{array}] \end{array}.$$

### 2.2. Tests of the Planar Actuator

**Figure 6:**
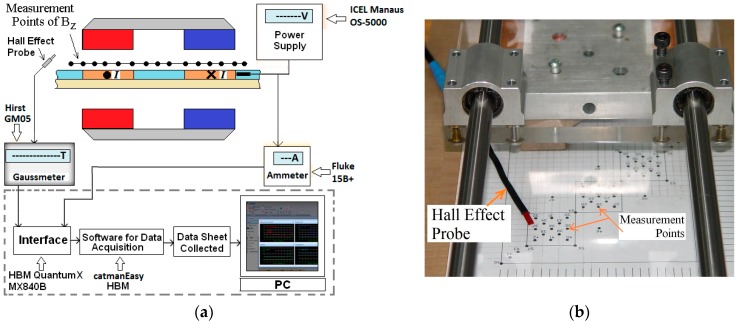
(**a**) Set-up for measurement of the *z*-component of the magnetic flux density vector; (**b**) details of the process of measurement.

The planar actuator was tested in order to compare its behavior with the results from the analysis. A test rig was designed. Measurements of magnetic flux density were carried out in the air-gap between the cars using a Gauss meter. For the tests, two cases were taken into account: measurement with no current in the armature phases; then with 3.0 A applied to phases located in between the permanent magnets. The results for the driving current were obtained by means of a digital ammeter. To guarantee correct correlation between magnetic flux density and current, a data acquisition system controlled by catman^®^Easy data acquisition software was employed providing good accuracy and correct triggering of the instruments. Figure 6a shows the set up for measuring the magnetic flux density and Figure 6b, a photograph with details of the process of measurement. A plastic template was employed to allow accurate placement of the Hall Effect probe on the coordinates defined for measurement of the magnetic flux density. The forces that act over the mover also depend on the distribution of flux density in the air-gap and that distribution is determined by the permanent magnet operation points and by the armature reaction.

**Figure 7:**
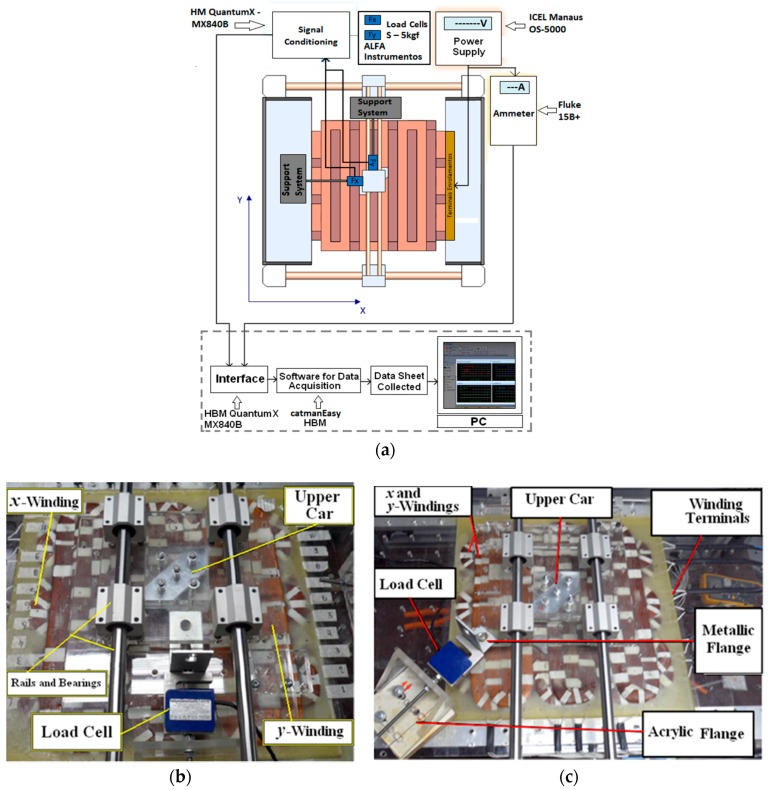
(**a**) Set-up for propulsion planar force; (**b**) photograph of the set-up with one load cell; (**c**) set-up of measurement of the resulting propulsion planar force when *x* and *y*-coils are fed by the same current simultaneously [8].

Measurements of the propulsion planar and normal forces under static conditions and of the attractive forces produced between the cars when they are misaligned, were taken using load cells [8]. Figure 7a presents the set-up for measurement of the static propulsion force that acts on the mover when the cars are aligned. As shown, each load cell is aligned horizontally with one car and is attached to it. Two load cells are presented in the picture with the aim of showing how each component of propulsion planar force is measured. However, each component can be measured using only one load cell at a time, according to Figure 7b that presents a photograph related to Figure 7a. In the last two figures, the polar surfaces of the four permanent magnets are aligned with one coil of each winding. When the *x*-coil is fed by current, a propulsion force tends to push the car through the *x*-axis. Hence, the mover applies a compressive force upon an S-type load cell. The rated capacity of the latter is 5 kgf. The output terminals of the load cell are connected to a signal conditioner. An ammeter measures the current in each coil. The output terminals of both instruments are connected to a computer. Again, catman^®^Easy data acquisition software was employed. For measurement of the total propulsion force that acts when one phase of each winding is fed by current, the load cell was positioned with its axis diagonally forming an angle of 45° with *x*-axis and *y*-axis, according to what is showed in Figure 7c [8].

The same set-up shown in Figure 7a can be used to measure the propulsion force when there is a displacement between the mover and an excited phase.

**Figure 8:**
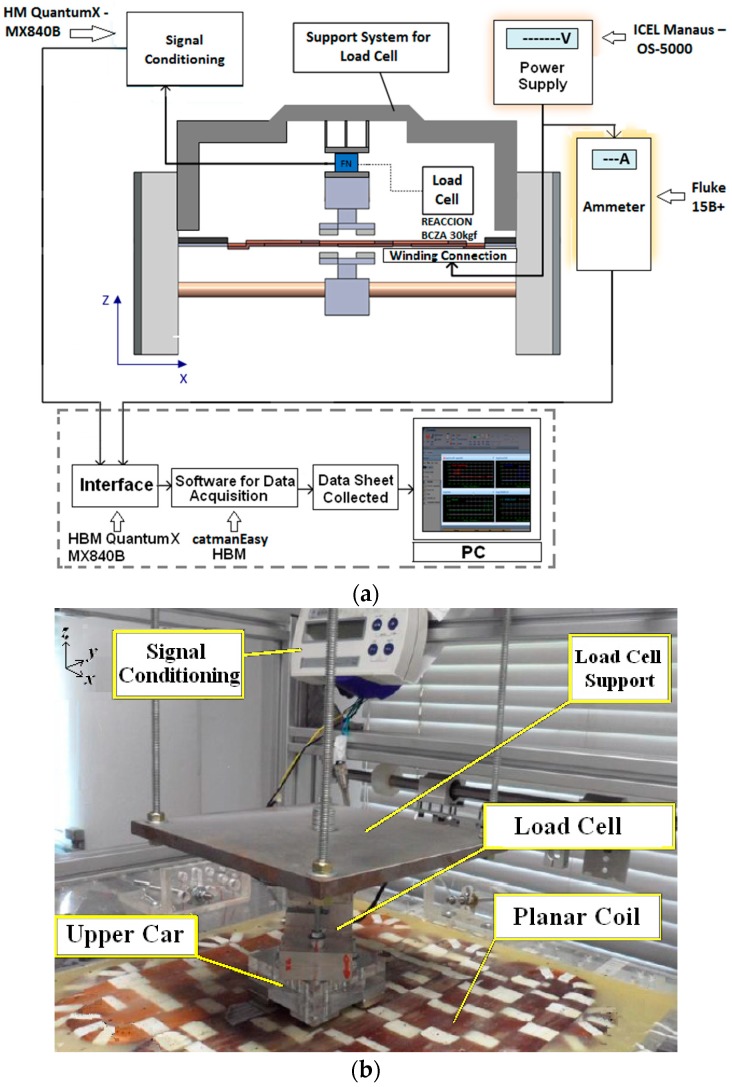
(**a**) Set-up for measurement of normal force; (**b**) photograph of the set-up.

For measurement of the normal force, Figure 8a, the load cell is aligned vertically between the upper car and a metallic plate rigidly attached to the suspension structure. Therefore, the car cannot develop movement during the tests. The S-type load cell is subjected to a traction force through the *z*-axis due to the attractive force between the permanent magnets of Cars *1* and *2*. The rated capacity of that load cell is 30 kgf. In both tests, measurements of propulsion and normal force were carried out within a current range from 0 to 3 A [8]. Figure 8b shows a photograph of the set-up for measurement of the normal force.

**Figure 9:**
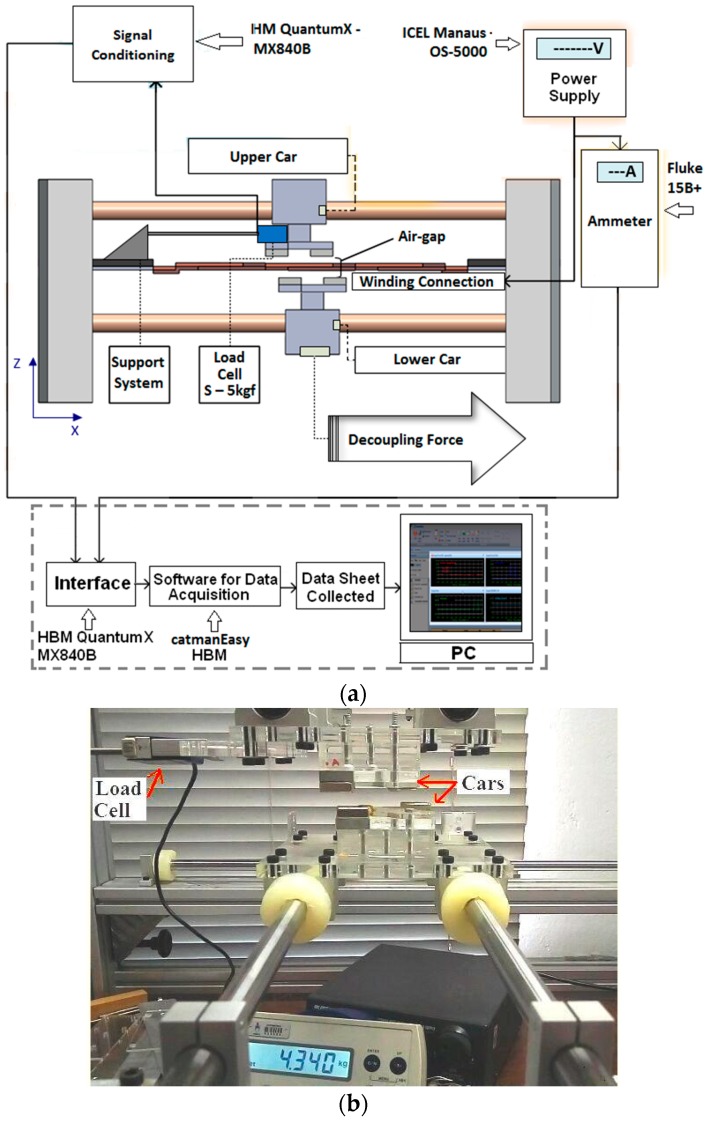
(**a**) Set-up of measurement of the linear stall force; (**b**) in the photograph, Cars *1* and *2* are misaligned accordingly [8].

The stall force is measured by means of an S-type load cell attached to the upper car and aligned horizontally with it through the *x*-axis. Linear displacements through the *x*-axis are applied to the lower car with respect to the upper car and the resulting values of force that act on the upper car are measured by means of the load cell. Figure 9a presents the set-up of measurement of the linear decoupling force and, in Figure 9b, the respective photograph. In Figure 9b, Cars *1* and *2* are misaligned, the displacement between them is equal to 12.5 mm and the armature windings are removed.

## 3. Results

**Figure 10:**
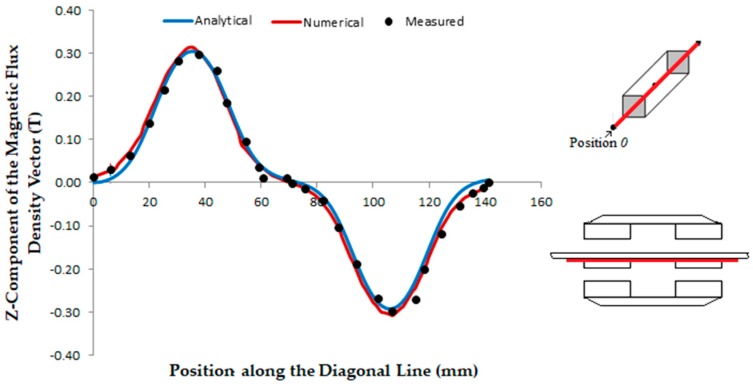
*Z*-component of magnetic flux density in the air-gap vs. the diagonal line when current in the phases is zero. The red lines in the actuator figures indicate the planes where values were taken.

Figure 10 shows the graph of the *z*-component of the magnetic flux density in the air-gap as measured on points of the plane *z* = 20 mm along the diagonal line when current in the phases is zero and the cars are aligned. Analytical results were obtained by means of (4). The horizontal axis of the graph represents the diagonal position in *x* and *y*-coordinates at each point, taken from position 0.

**Figure 11:**
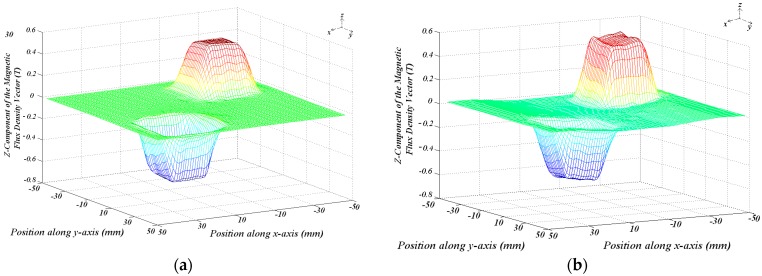
3D plotting of the *z*-component of the magnetic flux density: (**a**) with null current in the armature phases; (**b**) one *x*-phase is fed by a current of 3 A.

Figure 11a shows a 3D plot of the distribution of the *z*-component of the magnetic flux density vector measured in the air-gap on the plane *z* = 9.5 mm with zero current in the armature phases when the cars are aligned. Figure 11b presents the same plotting when an *x*-phase located between the permanent magnets of Cars *1* and *2* is fed by a current of 3 A.

**Figure 12:**
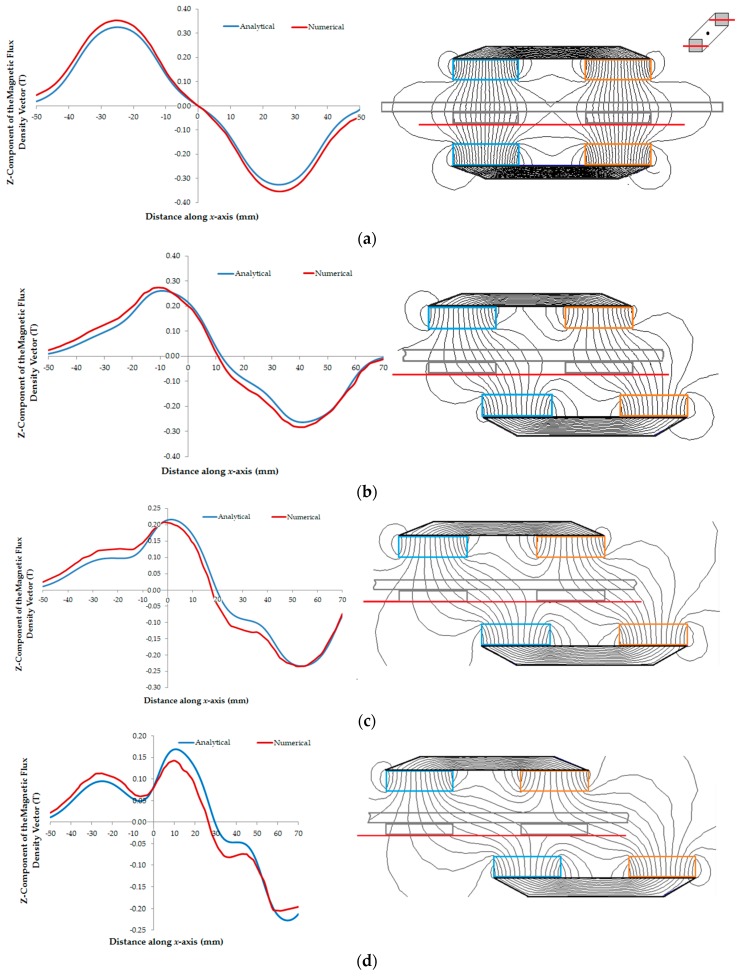
Graphs of the *z*-component of the magnetic flux density vector in the air-gap as a function of displacements between the cars and equal to: (**a**) 0 mm; (**b**) 20 mm; (**c**) 30 mm; and (**d**) 40 mm with null current in the phases.

The theoretical study of behavior of the magnetic flux density vector gave understanding of how the components of the vector acts when different displacements are applied to one car in relation to the other. Figure 12a–d present the graphs of the *z*-component of the magnetic flux density vector in the air-gap as a function of the distance for displacements equal to 0 mm, 20 mm, 30 mm and 40 mm, respectively. All graphs were calculated for points on the plane *z* = 16 mm and with zero current in the phases. The red lines in the actuator figures indicate the planes where the values of the magnetic flux density were taken. The analytical values of the *z*-component of magnetic flux density vector are calculated using (19). At the side of each graph, the picture shows the magnetic flux lines.

Table 1 presents the measured and theoretical values of the *z*-component of the magnetic flux density vector on the plane *z* = 20 mm related to Figure 10. Table 2 shows the same quantity related to the graphs of Figure 12 as function of that displacement between the cars. Positive and negative peak values of *B_gz_* are presented for the same displacement.

**Figure 13:**
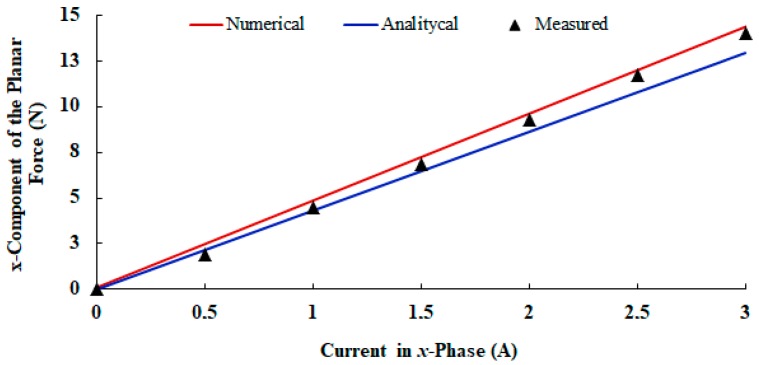
Planar force though *x*-axis vs. current in the *x*-phase located in between the permanent magnets of Cars *1* and *2*.

**Figure 14:**
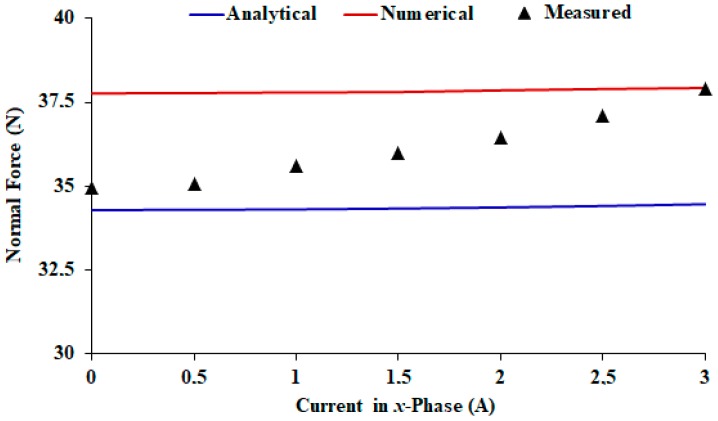
Normal force vs. current in the *x*-phase located in between the permanent magnets of Cars *1* and *2*.

Figure 13 shows the graph of the measured static propulsion force when only the *x*-phase located in between the permanent magnets of Cars *1* and *2* are fed with current. Figure 14 shows the respective normal force. In both graphs, numerical and analytical values are also presented. Numerical values were obtained by means of the 3D finite element model. Analytical results were calculated using (7) and (15), respectively. Table 3 shows a comparison between the theoretical and measured results of the normal force and of the average sensitivity calculated for the propulsion force.

**Figure 15:**
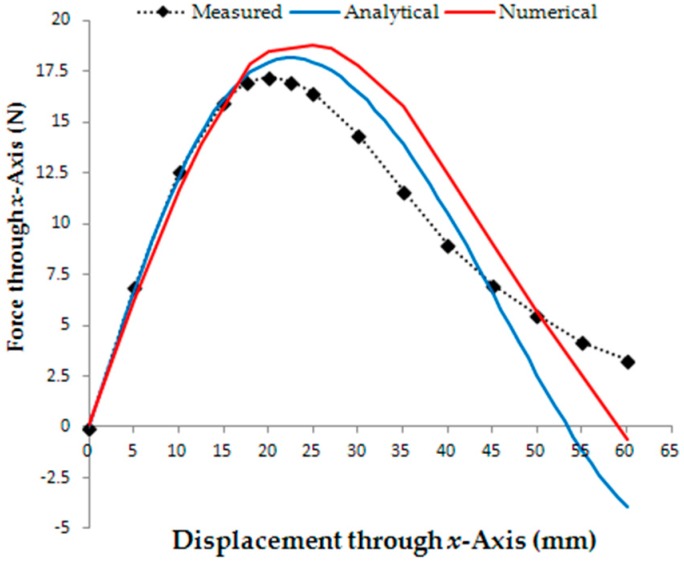
Stall force that acts through the *x*-axis to align Cars *1* and *2* as function of the displacement between them.

Figure 15 presents the results from tests of the linear force when the cars are misaligned from each other. Values of the linear force that acts through the *x*-axis to align the permanent magnets of the Cars *1* and *2* are presented as a function of the displacement between both cars through the *x*-axis. Analytical and numerical results are presented. The analytical results are calculated using (25). Table 4 shows the results of the linear force related to Figure 15.

**Figure 16:**
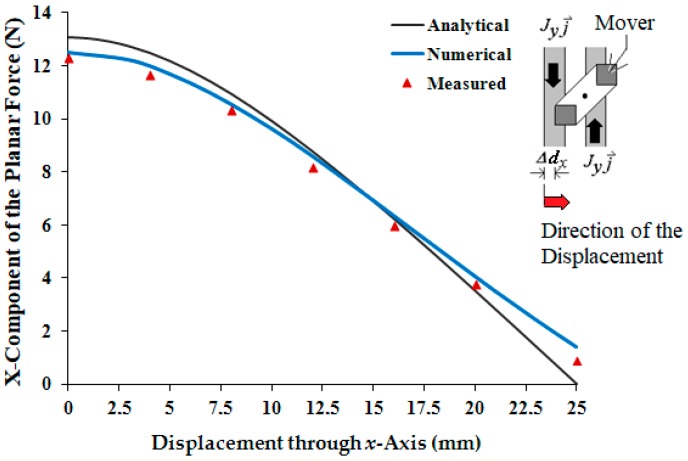
Graph of the *x*-component of the propulsion planar force vs. displacements through the *x*-axis of the mover with relation to an *x*-phase fed by current of 3 A.

The behavior of the *x*-component of the propulsion force is presented in Figure 16. This acts on the mover when there is a displacement through the *x*-axis between the mover and a phase current of 3 A. The theoretical values are also shown. The analytical results were obtained from (27).

## 4. Discussion and Conclusions

With the analysis and measurement of the magnetic flux density vector in the air-gap, it is possible to predict and understand the behavior of the electromagnetic forces. When both cars of the mover are aligned to each other, the magnetic flux density vector has a symmetric distribution in the air-gap when the windings are not fed by current. At plane *z* = 20 mm, the average measured *z*-component of *B_gz_* is 0.13 T and the respective peak value 0.30 T. The difference between the average measured and average analytical values of *B_gz_* and between the average measured and average numerical values is 7.69 %. There is no significant difference between measured and numerical peak values of *B_gz_*; the difference between the measured and analytical peak values is 3.33%. Hence, understood that these results can be considered in good agreement and, as such, they validate the analytical and numerical models. When the armature phase windings are excited, a non-uniform distribution of the magnetic flux density is observed, this is due to armature reaction.

The magnetic field produced by the armature winding currents is smaller when compared to the one produced by the permanent magnets, due to the long air-gap, that is, 24 mm and the maximum allowable value of the current, that is, 3 A. From the measured results shown in Figure 11, it is possible to observe the effect of the magnetic field produced by the armature windings on the distribution of the magnetic flux density in the air-gap. In Figure 11a, with no current in the armature phases, the distribution of the magnetic flux density in the air-gap is produced only by the permanent magnets on the mover and presents a symmetrical behavior with relation to the central line of the permanent magnets. The *z*-component of the magnetic flux density vector in the air-gap is equal to zero at *x* = 0 and *y* = 0 for any *z*-coordinate. When a phase located between the permanent magnets of Cars *1* and *2* is fed by current, the *z*-component of its magnetic flux density vector changes with relation to the *z*-component of the magnetic flux density vector produced by the permanent magnets. The resulting *z*-component in the air-gap does not have a symmetrical distribution with respect the central line of the permanent magnets; additionally, it presents a value different of zero at *x* = 0 and *y* = 0 for any *z*-coordinate within the air-gap. The distortion of the magnetic flux lines could provoke the magnetic saturation of one side of the ferromagnetic material of the back irons. However, due to the large air-gap and the rated value of the current, the magnetic field produced by the armature reaction is not enough to produce magnetic saturation in the ferromagnetic material of the actuator.

On the plane *z* = 9.5 mm, the value of *B_gz_* measured at a point that corresponds to the magnetic axis of the permanent magnets is 0.48 T when the current in a phase located between the permanent magnets of Cars *1* and *2* is 3 A. With no current, *B_gz_* is measured at the same point and is 0.47 T. For a diagonal line at plane *z* = 9.5 mm, which similar to the line in Figure 10, the average measured value of *B_gz_* is 0.19 T with no winding current and 0.18 T when the current in a *x*-phase located in between the cars is 3 A. For the same plane, numerical and analytical results of *B_gz_* gave good agreement in relation to the measured results: the differences are 5.84% and 5.41% for the average of analytical values, with 0 and 3 A current, respectively. With respect to the average numerical results, the differences are 1.05% and 11.19%, for the same values of current.

When the cars are aligned, the measured sensitivity of the propulsion planar force, with only the *x*-phase fed by current, is 4.48 N/A. For a 3-A current, the propulsion planar force that acts on the mover through an axis is 13.44 N. As shown in Table 3, the theoretical and measured results show good agreement with each other. These results also validate the analytical and numerical models for propulsion force calculation. The same can be said for the normal force, since numerical and analytical results present differences smaller than 10%. The measured values of normal force are equal to 34.9 N (*I* = 0) and 37.9 N (*I* = 3 A) when there is only an *x*-phase current. The mechanical support, provided by a set of linear guides and bearings was designed using the total attractive normal force that acts between the cars when phases of *x* and *y*-windings are excited.

The misalignment between the cars causes a non-uniform distribution of the magnetic flux density in the air-gap. This can be verified in Table 2; the longer the displacement between the cars, the higher the difference between the positive and negative peaks of *B_gz_*. In Figure 12b–d, it is possible to observe the significant distortion of the magnetic flux lines in the air-gap due to the displacement between the cars. In Figure 15, the *x*-component of the linear force that tends to align the cars is presented as a function of the displacement. The maximum measured value of this force is equal to 17.26 N when the displacement is equal to 20 mm. From this result, it is possible to conclude that the linear force necessary to produce the magnetic decoupling between the cars must be higher than 17.26 N. It can also be concluded that the propulsion planar force produced when only one phase winding is excited is not sufficient to cause a misalignment of the cars with respect to each other. The propulsion force produced, when a phase is fed by 3 A, is equal to 13.44 N. When both windings, *x* and *y*, are fed with 3 A, the resulting propulsion force in a diagonal line over the plane is equal to 19 N and the maximum linear force that tends to keep the cars coupled is equal to 24.41 N.

Figure 16 shows the behavior of the propulsion planar force as a function of the displacement between the mover and an excited phase for a current of 3 A. As expected, the force tends to zero, when the displacement tends to 25 mm. A displacement that is higher than this value causes the inversion of the direction of the force.

The numerical analysis and the analytical model are good tools for simulating the static behavior of the planar actuator with respect to the magnetic flux density and the forces that act on the cars. The theoretical results show good agreement with the measurements. According to what was reached in the results for the magnetic flux density, the analytical and the numerical models produced results with some perceptual differences between them and with relation to the measured values. In Table 1, the analytical and measured peak values of the *z*-component of the magnetic flux density vector are similar, while the numerical value is 3.33% higher than the measured one. In the same table, analytical and numerical average values of *B_gz_* present a higher difference. By means of Figure 10, it is possible to observe that theoretical and measured shapes of the distribution of the magnetic flux density are in good agreement in the region between Cars *1* and *2*. In the points out of this region, there is a slight divergence between the shapes. Where there is a predominance of the air in the path of the magnetic flux, the theoretical models can present results of magnetic flux density with major errors, principally the analytical model. That effect can be seen in Figure 12. The longer the displacement between the cars, the bigger the differences in the numerical and analytical shapes of the distribution of magnetic flux density, because the path of the magnetic flux involves a higher quantity of air. That characteristic can have influence on the measurement of forces, since they depend on the distribution of the magnetic flux density produced by the permanent magnets and by the current in the windings, mainly in the analysis of the stall force. In Figure 15, the difference in the results of the stall force is higher with the increasing displacement of the cars since the analytical model depends on a more widespread magnetic path through the air that can be troublesome to set. In general terms, the results present a good agreement and are adequate to aid the analysis, the design and the operation the planar actuator.

## Figures and Tables

**Table 1:** Measured and theoretical values of the *z*-component of the magnetic flux density vector on the plane *z* = 20 mm related to Figure 10.

*Z*-Component of the Magnetic Flux Density Vector				
	Average Value (T)	Difference (%)	Peak Value (T)	Difference (%)
Measured	0.13	-	0.30	-
Analytical	0.12	−7.69	0.30	0
Numerical	0.14	7.69	0.31	3.33

**Table 2:** Theoretical values of the *z*-component of the magnetic flux density vector at plane *z* = 16 mm, shown in Figure 1, in between Cars *1* and *2*, as a function of the displacement between Cars *1* and *2*.

	Displacement (mm)	Peak Values	
		*B_gz_* (T)	−*B_gz_* (T)
Analytical	0	0.33	−0.33
	20	0.26	−0.26
	30	0.22	−0.23
	40	0.17	−0.23
Numerical	0	0.35	−0.35
	20	0.28	−0.28
	30	0.21	−0.23
	40	0.11	−0.14

**Table 3:** Theoretical and measured results of propulsion and normal forces.

	Propulsion Force		Normal Force			
	Sensitivity (N/A)	Difference (%)	*I* = 0	Difference (%)	*I* = 3 A	Difference (%)
Measured	4.48	-	34.9	-	37.9	-
Analytical	4.31	−3.79	34.3	−1.72	34.5	−8.97
Numerical	4.78	6.70	37.8	8.31	37.9	0

**Table 4:** Theoretical and measured results of the maximum linear force that acts through the *x*-axis to align Cars *1* and *2* as function of the displacement between them.

	Displacement (mm)	Maximum Linear Force (N)	Difference (%)
Measured	20	17.26	
Analytical	22	18.15	5.16
Numerical	25	18.78	8.81

## Appendix A

Table A1 presents the main characteristics of the double-sided moving-PM-type planar actuator based on orthogonal planar windings [8].

**Table A1:** Characteristics of the planar actuator [8].

Item	Feature
air-gap length, *l_g_* winding number	24 mm 2 (*x*- and *y*-windings)
number of phases per winding	6
number of coils per phase	1
number of turns per phase	280
coil width, *l_b_*	4 mm
armature area	150 (L) × 150 (W) mm
coil resistance	26.6 Ω
rated current	3 A
back iron material	SAE 1045 Steel
pole pitch	50 mm
PM number	4
PM material	NdFeB
PM dimensions	25 (*w_m_*) × 25 (*w_m_*) × 8 (_lm_) mm
PM remanence	1.19 T
PM intrinsic coercitivity	1018 kA/m
PM normal coercitivity	883 kA/m
distance on *xy*-plane between two permanent magnets of the same car through *x* and *y*-axes, 2*l_d_*	25 mm
distance along *z*-direction between the two back irons, *l*	40 mm
distance along *z*-direction between the back iron of Car *1* and the *x*-winding, *l_c_*	16 mm
distance along *z*-direction between the back iron of Car *1* and the *y*-winding, *l_w_* − *l_b_*	20 mm
